# Metaphyseal Sleeve Failure in Revision Total Knee Arthroplasty

**DOI:** 10.7759/cureus.18054

**Published:** 2021-09-17

**Authors:** Theodoros Bouras, Peter Fennema, Rhidian Morgan-Jones, Sanjeev Agarwal

**Affiliations:** 1 Department of Trauma & Orthopaedics, Cardiff & Vale University Health Board, University Hospital Llandough, Cardiff, GBR; 2 Epidemiology and Public Health, AMR Advanced Medical Research, Männedorf, CHE

**Keywords:** contact zones, implant survival, porous coated, metaphyseal sleeves, revision total knee arthroplasty

## Abstract

Introduction

A significant percentage of patients require re-revision surgery regardless of the demonstrated durable short- and mid-term clinical results using metaphyseal sleeves in revision total knee arthroplasty (TKA). The aim of this study was to identify the association between sleeve alignment and contact zones, with loosening in patients with revision TKA.

Materials & Methods

Of a series of 103 patients who underwent revision TKA, at a mean follow-up of eight years, six patients were re-revised for tibial loosening. These patients were compared with 19 unrevised control subjects in a 1:3 ratio. We calculated and compared the cumulative number of contact zones between the porous-coated part of the sleeve and bone on immediate postoperative X-rays between re-revised and unrevised patients. The main hypothesis was that neutral positioning and absolute circumferential contact between trabecular metaphyseal bone and porous-coated part of the sleeve would lead to a better outcome.

Results

The use of a conservative (nonparametric) approach indeed revealed better circumferential contact between trabecular metaphyseal bone and porous-coated part of the sleeve among the survivors, i.e., survivors: median (interquartile range [IQR]): 3 (2-4); failures: 3 (1-3), *p* = 0.003 (Mann-Whitney [MW] test). The difference was borderline significant for coronal alignment, i.e., survivors: median (IQR): −1 (−4 to 2); failures: 0 (−1 to 3), *p* = 0.0569 (MW test).

Conclusion

A circumferential bony contact of the metaphyseal sleeve would lead to better survival of the revision implant, whereas the degree of varus fixation did not seem to influence the longevity of the implant.

## Introduction

The need for revision total knee arthroplasty (RTKA) is constantly increasing over the years. The projected estimates for England and Wales suggest an increase in the volume of RTKA by 332% by the year 2030 [1]. In the challenging task of reconstruction and fixation in RTKA, especially in the presence of bone defects, the concept of zonal fixation provides the surgeon a working methodology toward the achievement of best results and lower re-revision rates [2,3]. The use of porous-coated metaphyseal sleeves is a modern technique addressing bone deficiency. It creates a stable platform for the femoral and/or tibial components, offering long-term biologic fixation to host bone. Reliable stability of the metaphyseal construct decreases torsional and shear stresses at the cement-bone interface [4]. Recent evidence suggests that the use of metaphyseal sleeves demonstrates excellent short-, mid-, and long-term clinical results and radiographic fixation [5-15]. Different modes of constraint, or sleeve and stem fixation, such as cemented or cementless, do not appear to impact the survivorship and the osseous integration of the porous-coated part of the metaphyseal sleeve. This has deprived the literature of studies looking specifically for failure reasons of the metaphyseal sleeve fixation. Proper assessment of indications and contraindications, as well as accurate surgical technique, is of utmost importance. The hypothesis of this study was that neutral positioning and complete circumferential contact between trabecular metaphyseal bone and porous-coated part of the sleeve would lead to a better outcome.

## Materials and methods

**Figure 1:**
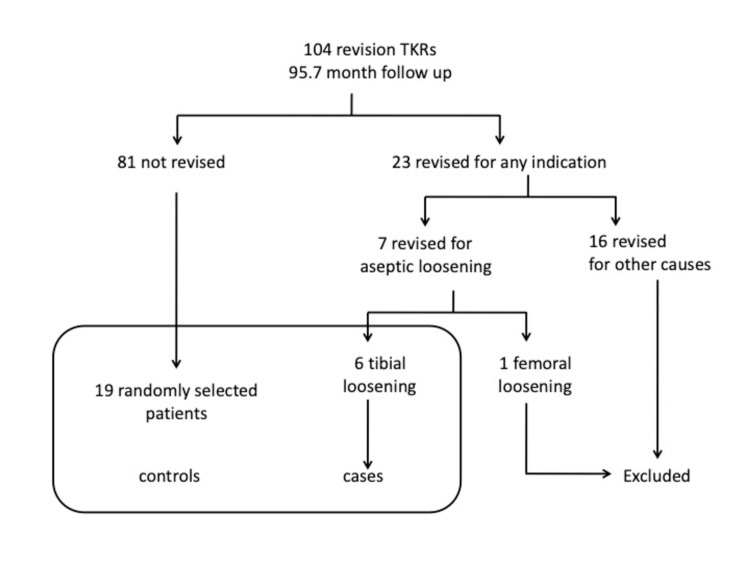
Case-control population study group. TKR, total knee replacement.

A retrospective review of a prospectively maintained longitudinal revision total knee arthroplasty (TKA) database was performed. There were 103 patients (104 knees) identified as having undergone revision TKA using porous-coated metaphyseal sleeves (DePuy, Warsaw, Indiana) [8]. At a mean follow-up of 95.7 months, there were 23 (22.1%) re-revisions for any reason. All patients who had index revision and re-revision for infection, stiffness, instability, peri-prosthetic fracture, or undiagnosed pain were excluded from the 23 cases, 6 patients (26%) were re-revised for tibial loosening, and 1 (4.3%) for femoral loosening. All six cases of aseptic tibial loosening were included in the study. The patient with femoral loosening had disengagement at the stem sleeve junction and was not included in the study as seen in Figure 1.

Control patients were sampled in a 1:3 ratio among the patients with similar follow-up but without loosening. Two adult reconstructive knee orthopedic surgeons (one consultant and one senior fellow) independently assessed and compared the immediate postoperative X-rays, using a digital viewing platform, at the time of index revision, between the six tibial re-revised patients and a group of 19 patients that had maintained the sleeve in situ. Radiographs included standard weight-bearing anteroposterior (AP) and lateral views of the knee and were undertaken based on a standard protocol by experienced radiographers. The degree of coronal plane alignment and the porous-coated part of the sleeve was assessed. This assessment was based on the presence of radiolucency at the metal bone interface of the metaphyseal sleeve. On the tibial side, only the proximal third of the sleeve is porous-coated. This was the zone of interest for metal-bone contact.

**Figure 2:**
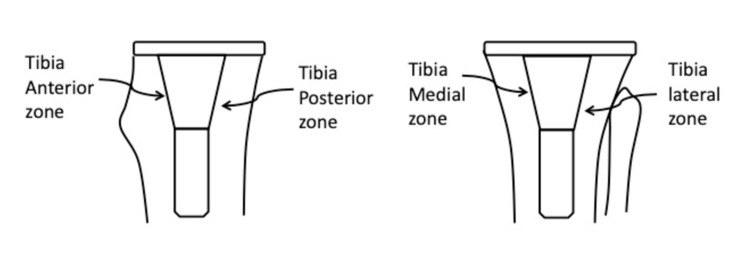
The zones used for the scoring system.

The two standard radiographic views provided four regions of interpretation: anterior and posterior on the lateral view; and medial-lateral on the AP view (Figure 2).

Absolute contact between trabecular metaphyseal bone and porous-coated part of the sleeve in each region was graded as “1” and any gap in the bone-sleeve interface as “0.” A total score of “4” meant absolute press-fit circumferential contact between trabecular metaphyseal bone and porous-coated part of the sleeve. This score is similar to methods used to quantify fracture healing. The degree of varus or valgus fixation of the sleeve was also measured in all patients. We did not routinely use CT scans for assessing osseointegration or loosening in our patients. This was a retrospective study and no patient intervention or contact was involved. Formal ethical approval was not required.

Statistical analysis

Statistical analyses were performed using Stata 15.1 software (StataCorp, College Station, Texas). Continuous data are presented as mean ± standard deviation (range); the Mann-Whitney (MW) test was used to compare between-group differences. Categorical variables are presented as frequencies, and Fisher's exact test was used to determine between-group differences. Two-sided *p*-values of 0.05 were deemed to indicate statistical significance.

## Results

Baseline characteristics of cases and controls are summarized in Table 1.

**Table 1:** Baseline characteristics of cases and controls.

	Patients with tibial loosening (cases)	Patients without tibial loosening (controls)	*p*-value
Sample size	6	19	
Age at inclusion (years)	72.0 ± 1.7	77.7 ± 1.7	0.056
Sex (female:male)	2:4	7:12	0.637

The radiological scores of patients in the study group and control group are summarized in Table 2.

**Table 2:** Radiological scores of study group and control group.

Score	Study group (number of patients)	Control group (number of patients)
0		
1	1	
2	2	3
3	3	2
4		14

**Figure 3:**
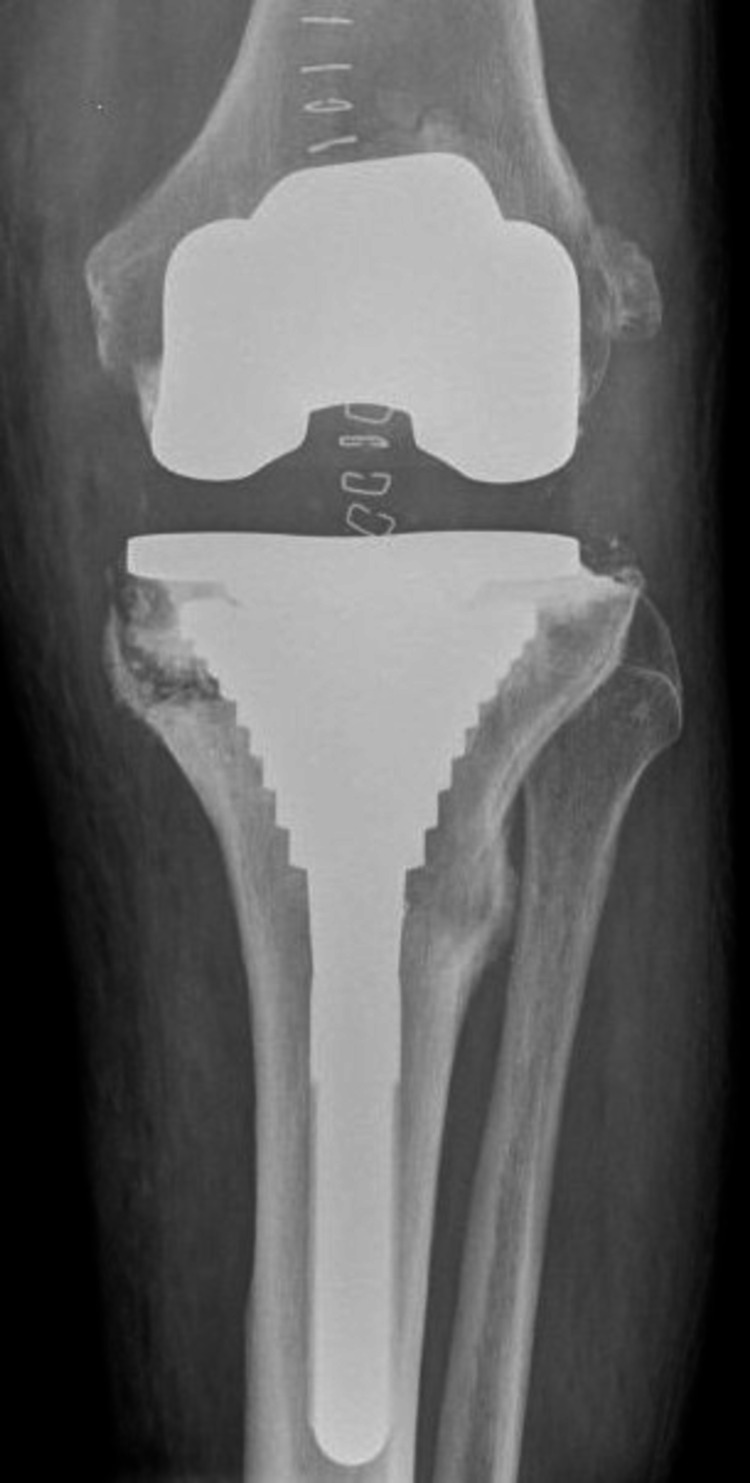
Early postoperative X-ray anteroposterior view showing lack of metal-bone contact in the medial aspect of the metaphyseal sleeve.

**Figure 4:**
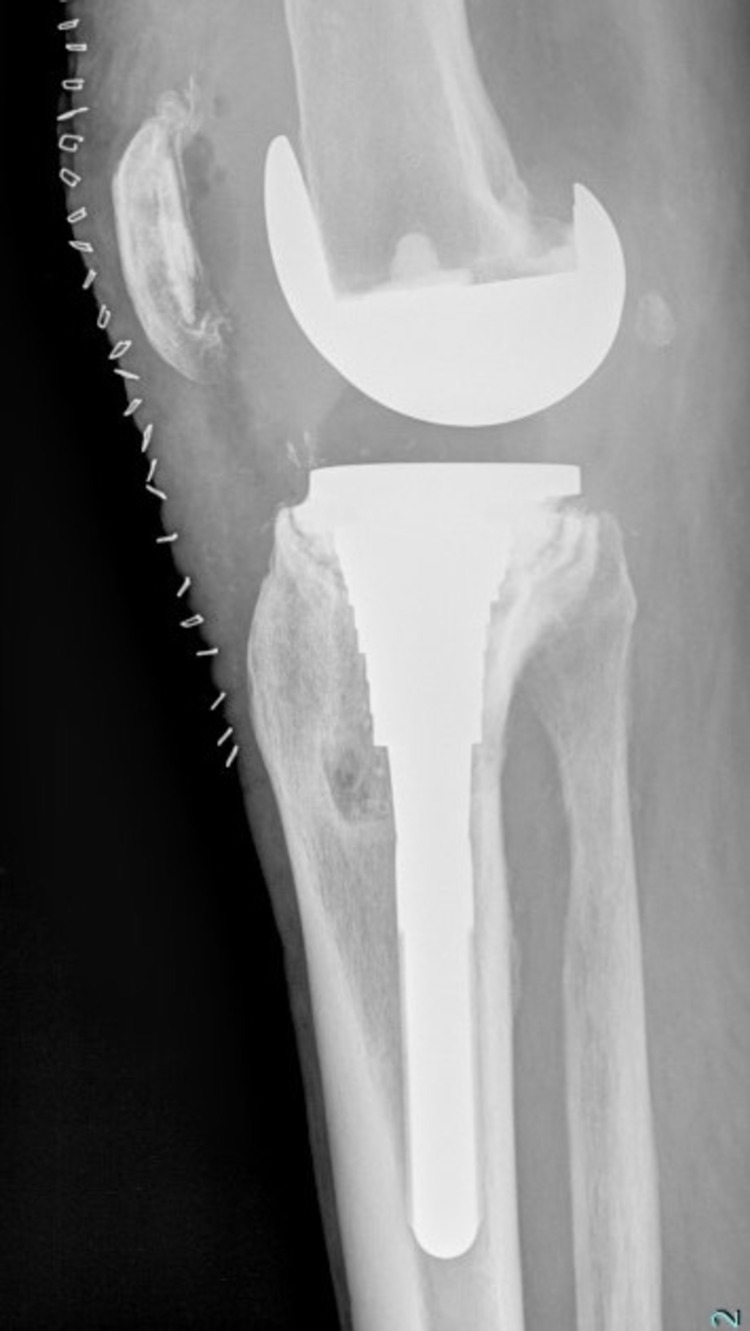
Early postoperative X-ray lateral view showing lack of metal-bone contact in the anterior and posterior aspect of the metaphyseal sleeve.

**Figure 5:**
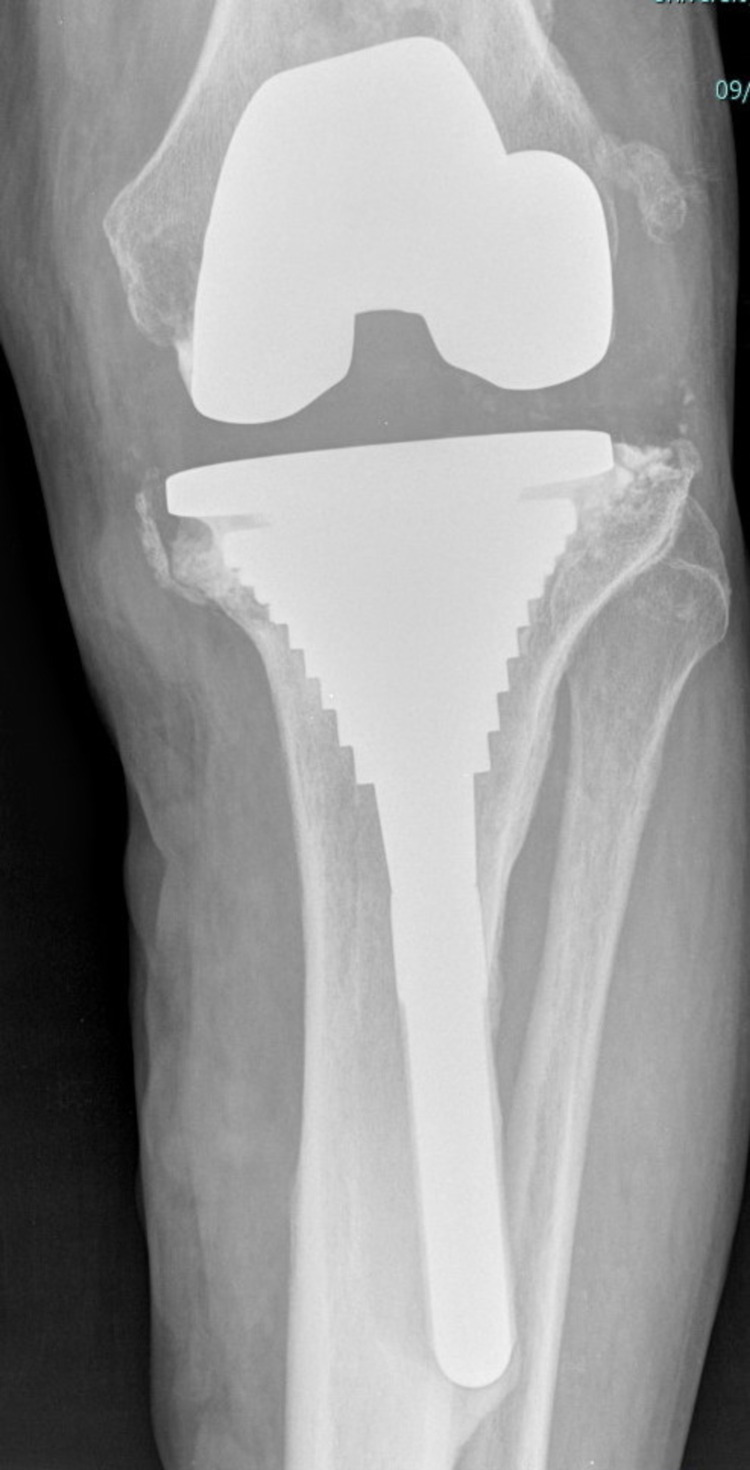
A 72-month follow-up X-ray anteroposterior view. The tibial component has loosened and migrated into varus.

**Figure 6:**
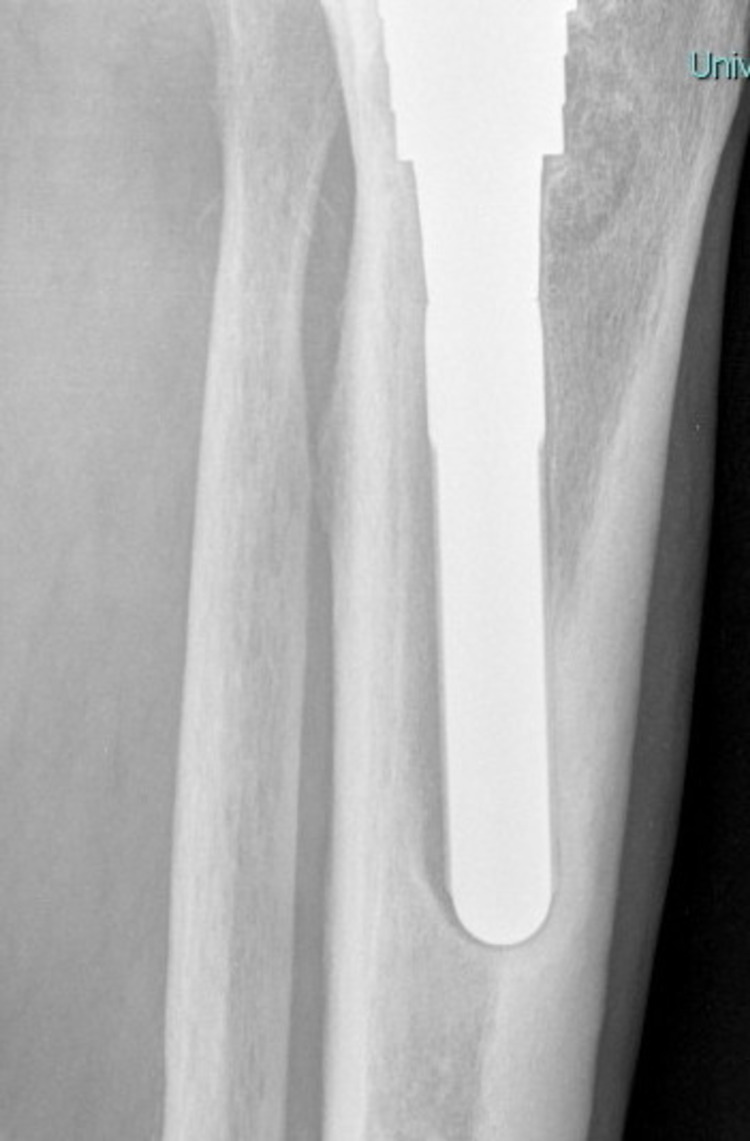
A 72-month follow-up X-ray lateral stem tip view. The tibial component has loosened and migrated into varus.

None of the six re-revised patients (study group) demonstrated absolute press-fit circumferential contact between trabecular metaphyseal bone and porous-coated part of the sleeve. Three out of six (50%) scored “3” with inadequate contact in the medial (AP) region of the sleeve while two patients (33.3%) scored “2.” In one patient (16.7%), absolute contact was seen only in the lateral region of the sleeve (fixation score 1), which finally collapsed in varus (Figures 3-6).

From the control group, there were 14 patients (73.7%) who had absolute contact in all four regions. A gap in the anterior interface was seen in two patients (10.5%). The remaining three patients (15.8%) scored “2.” Alignment between 3° varus and 1° valgus was found in the six re-revised cases. The alignment for the unrevised patients was between 1° varus and 4° valgus. Taking a conservative (nonparametric) approach, better circumferential contact between trabecular metaphyseal bone and porous-coated part of the sleeve among the survivors was found, i.e., survivors: median (interquartile range [IQR]): 3 (2-4); failures: 3 (1-3), *p* = 0.003 (MW test). For coronal alignment, the difference did not reach the significance threshold of 0.05, i.e., survivors: median (IQR): −1 (−4 to 2); failures: 0 (−1 to 3), *p* = 0.0569 (MW test).

## Discussion

Achieving secure long-lasting fixation of the implant in the bone is an important component of revision knee replacement. Both cemented and cementless fixations principles exist in order to achieve this goal. Maximizing contact with host bone is desirable in either fixation method. The most important finding of this study was that a circumferential bony contact of the porous-coated part of the metaphyseal sleeve would lead to better survival of the revision implant, whereas the degree of varus fixation did not seem to influence the longevity of the implant. A press-fit insertion of the sleeve would require sequential broaching with progressively increasing sizes until axial and rotational stability is achieved [4]. Depending on the bone loss, impaction grafting or morselized allograft could be used to fill the remaining defects [16]. We did not use allograft in any of our patients and are unable to comment on the impact of graft usage and sleeve contact with host bone, or its influence on the long-term survival of the sleeve. Bone coverage of uncemented sleeves, with a minimum of 75%, to achieve optimal osseointegration was reported by studies included in a recent systematic review [17]. Another one reported a high osseointegration rate demonstrated by the use of metaphyseal sleeves [18]. A recent case series reported proximal radiolucent lines in nine tibial sleeves, one year following revision surgery compared to immediate postoperative X-rays. These lines had not progressed in the five-year follow-up and the sleeves did not require revision. In the same study, 12 tibial sleeves had subsided >1 mm compared to the original postop X-ray, without further progression and subsequent re-revision surgery [5]. Another recent study reported minor radiolucent lines detectable in a few sleeves but no implant migration [9]. A retrospective cohort of osseointegrated sleeves reported no radiolucencies in the porous coated part of the sleeves at the five-year follow-up. Despite the well-fixed implants, radiolucencies were seen in the uncoated part of the sleeve. This was attributed to possible pivoting especially in combination with long diaphyseal stems [6]. Ihekweazu et al. in 2019 published a retrieval analysis study reporting bone ongrowth coverage of the entire porous surface of the tibial sleeve on average of 14.7 ± 3.4%. The authors correlated the radiological findings with the bone ongrowth in the retrieved specimens suggesting that plain radiology was highly unreliable to detect biologic fixation [19]. In our study, the coronal alignment for the six revised cases was between 3° varus and 1° valgus, and for the unrevised patients was between 1° varus and 4° valgus. This was borderline significant to the final outcome. Martin-Hernandez et al. reported a similar outcome with satisfactory alignment between 3° varus and 3° valgus [10]. With the relatively small number of patients, we are unable to comment about contact of specific aspects-medial/lateral/anterior/posterior-of the sleeve, which would influence osseointegration and stability. A malaligned sleeve, loaded more on one side, couples with lack of osseous contact on the same side can lead to cantilever failure as shown in Figures 3-6. The current literature on metaphyseal sleeves is mainly comprised of case series with limited information on failure mechanisms; hence, our study adds to the body of evidence on the subject. However, this is limited by the small sample size, which decreases the options such as a stratified or multiple logistic regression analysis to assess the presence of confounding.

## Conclusions

Absolute circumferential bony contact of the porous-coated part of the metaphyseal sleeve at the time of index revision TKA surgery can lead to better implant survival. Coronal alignment does not seem to play a significant role in the final outcome.
